# Supplementary material for: South Pacific sea surface temperature and global ocean circulation changes since the late Miocene
Source: Nat Commun. 2025 Jul 17;16:6593. doi: 10.1038/s41467-025-62037-w (PMC12271382; doi:10.1038/s41467-025-62037-w)
Supplement: Supplementary file 1 — Supplementary Information [file 41467_2025_62037_MOESM1_ESM.pdf]

Supplementary Information for

South Pacific sea surface temperature and global ocean circulation changes since the late Miocene

by

Antje Wegwerth[1*], Helge W. Arz[1], Jérôme Kaiser[1], Gisela Winckler[2], Lester Lembke-Jene[3], Vincent Rigalleau[3], Nicoletta Ruggieri[3], Henrik Sadatzki[3,4], and Frank Lamy[3]

[1]Leibniz Institute for Baltic Sea Research Warnemünde (IOW), Marine Geology, Rostock, Germany
[2]Columbia University, Lamont-Doherty Earth Observatory, Palisades, United States
[3]Alfred Wegener Institute, Helmholtz Center for Polar and Marine Research, Bremerhaven, Germany
[4]MARUM-Center for Marine Environmental Sciences, University of Bremen, Bremen, Germany

This pdf file includes Supplementary Figures S1-S4.

[*] Corresponding author: Antje Wegwerth
e-mail/phone: antje.wegwerth@io-warnemuende.de / +49-381-5197-3481

Supplementary Figures

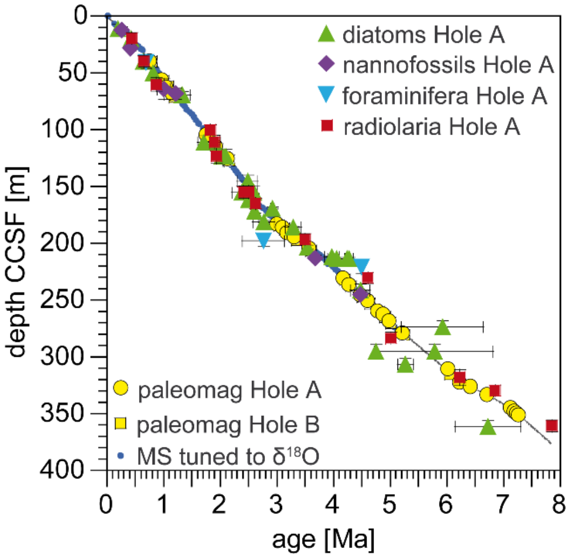

Supplementary Figure 1: Stratigraphy of IODP383 site U1543. Age model of IODP383 site U1543 based on biostratigraphic age control points compared to the age model using magnetic susceptibility tuned to the LR04 δ^{18}O-stack[1] (0-5 Ma; modified after Lamy et al.[2]) and paleomagnetic data (5-7.8 Ma).

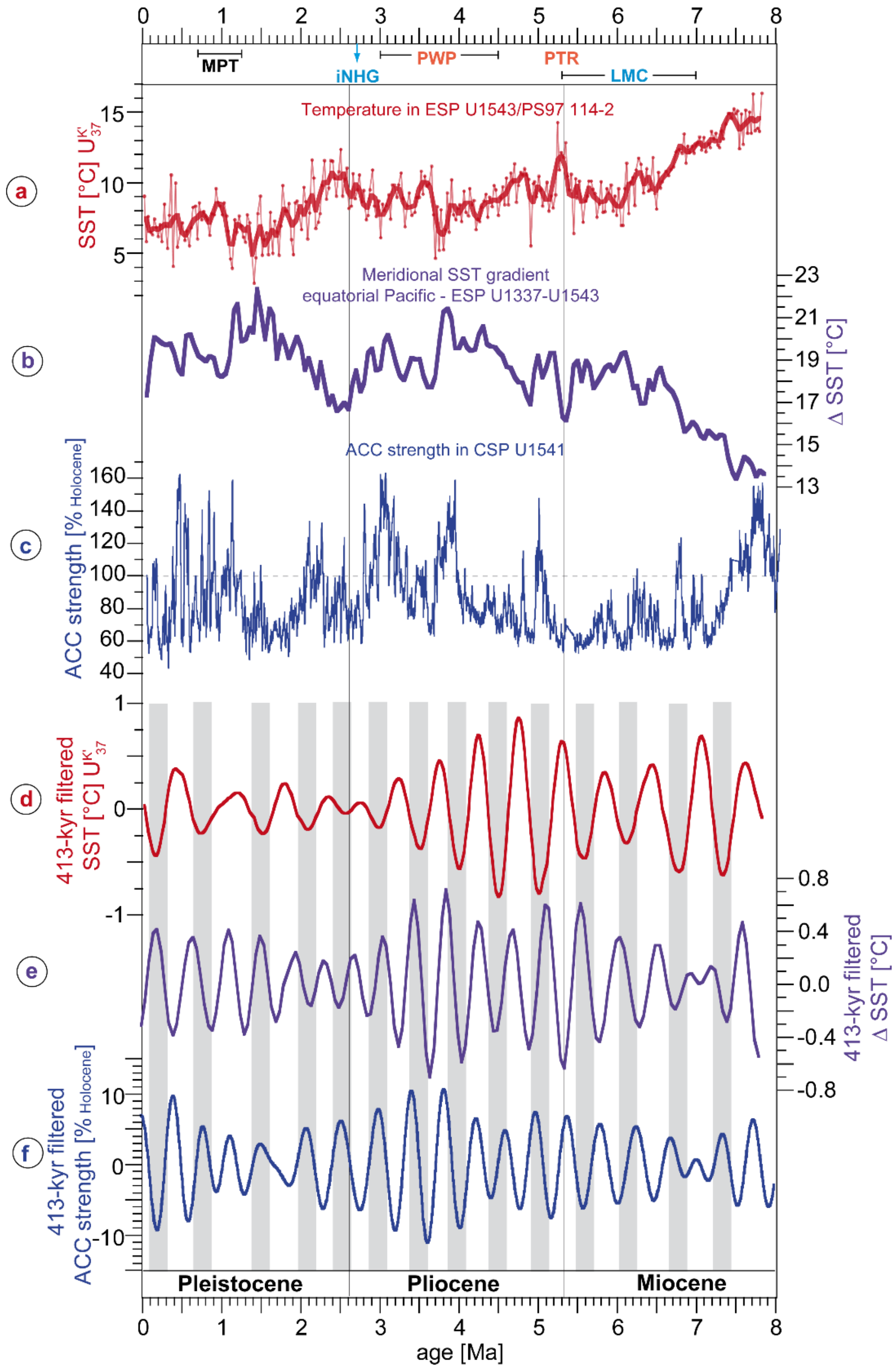

Supplementary Figure 2: Long-term variations in temperature and ACC since the late Miocene. Temporal variation of **a)** alkenone-based sea surface temperatures in the Eastern South Pacific Ocean ($SST_{UK'37}$; U1543/PS97-114-2; this study), **b)** meridional SST gradients between the eastern equatorial Pacific Ocean[3] (ODP1337) and the Eastern South Pacific Ocean (U1543, this study), **c)** strength of the Antarctic Circumpolar Current (ACC) in the Central South Pacific Ocean based on Zr/Rb ratios of site U1541 (Lamy et al.[4]; 5.3-8 Ma this study), **d)** filtered SST with Gaussian band-pass filter centred at 413 kyr, **e)** filtered SST gradient record at 413 kyr, and **f)** filtered ACC record at 413 kyrs[4]. Grey vertical bars denote periods of cooling. LMC: late Miocene cooling; PTR: Pliocene global temperature reversal; PWP: Pliocene warm period, iNHG: intensification of the Northern Hemisphere Glaciation, MPT: mid-Pleistocene transition.

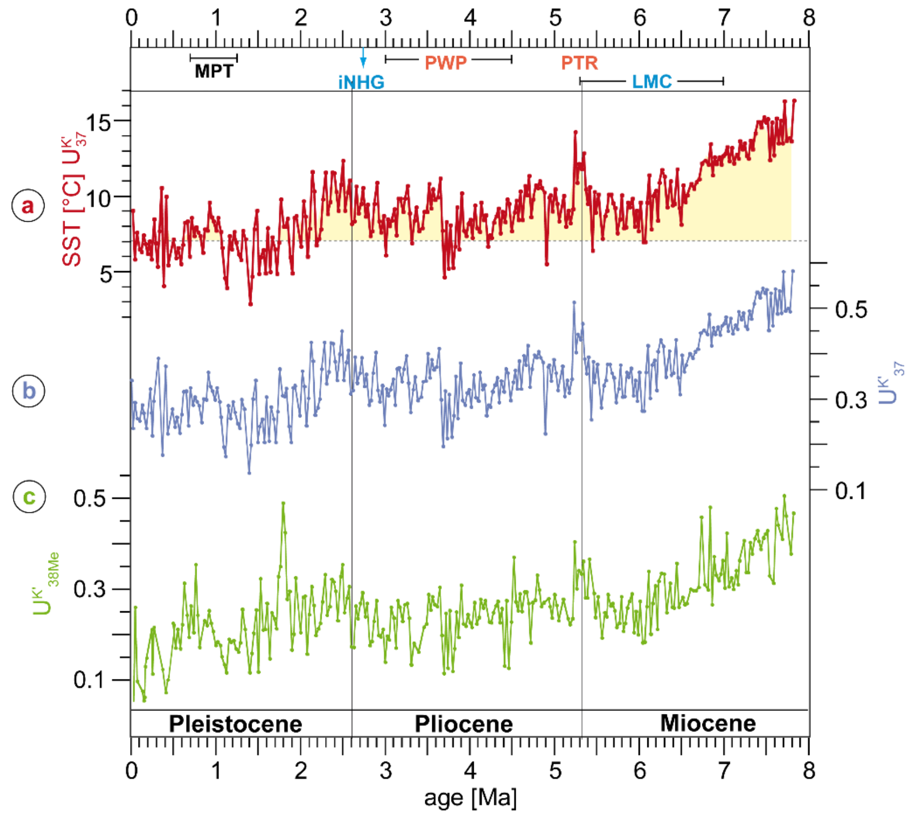

Supplementary Figure 3: Long-term variations in sea surface temperature and alkenones in the Eastern South Pacific Ocean ($SST_{UK'37}$; U1543/PS97-114-2; this study). Temporal variation of **a)** alkenone-based sea surface temperatures (yellow shading denotes warmer than modern conditions); **b)** the $U^{K'}_{37}$ index; and **c)** the $U^{K'}_{38Me}$ index. LMC: late Miocene cooling; PTR: Pliocene global temperature reversal; PWP: Pliocene warm period, iNHG: intensification of the Northern Hemisphere Glaciation, MPT: mid-Pleistocene transition.

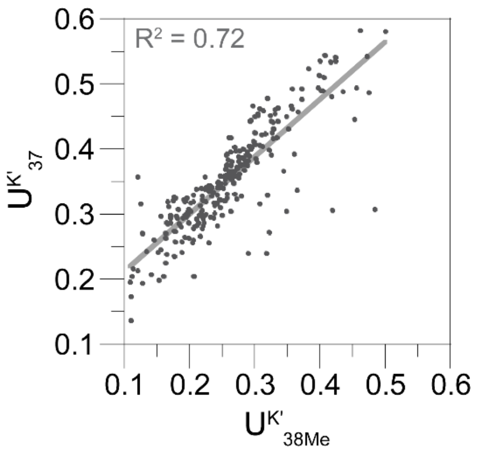

Supplementary Figure 4: Correlation between different alkenone unsaturation indices from samples of IODP 383 site U1543. Positive linear correlation between the $U^{K'}_{37}$ and the $U^{K'}_{38Me}$ indices supporting that the $U^{K'}_{37}$-paleothermometer was not affected by potential community shifts[5,6].

Supplementary references

1. Lisiecki, L.E., Raymo, M.E., 2005. A Pliocene-Pleistocene stack of 57 globally distributed benthic δ18O records. Paleoceanography 20, DOI:10.1029/2004PA001071.
2. Lamy, F., Winckler, G., & Alvarez Zarikian, C. A. (2021). Volume 383: Dynamics of the Pacific Antarctic Circumpolar Current (DYNAPACC) (Expedition 383 Scientists, Ed.; Vol. 383). International Ocean Discovery Program. DOI:10.14379/iodp.proc.383.2021.
3. Liu, J., Tian, J., Liu, Z., Herbert, T.D., Fedorov, A.V., Lyle, M., 2019. Eastern equatorial Pacific cold tongue evolution since the late Miocene linked to extratropical climate. Science Advances 5, eaau6060, DOI:10.1126/sciadv.aau6060.
4. Lamy, F., Winckler, G., Arz, H.W., Farmer, J.R., Gottschalk, J., Lembke-Jene, L., Middleton, J.L., van der Does, M., Tiedemann, R., Alvarez Zarikian, C., Basak, C., Brombacher, A., Dumm, L., Esper, O.M., Herbert, L.C., Iwasaki, S., Kreps, G., Lawson, V.J., Lo, L., Malinverno, E., Martinez-Garcia, A., Michel, E., Moretti, S., Moy, C.M., Ravelo, A.C., Riesselman, C.R., Saavedra-Pellitero, M., Sadatzki, H., Seo, I., Singh, R.K., Smith, R.A., Souza, A.L., Stoner, J.S., Toyos, M., de Oliveira, I.M.V.P., Wan, S., Wu, S., Zhao, X., 2024. Five million years of Antarctic Circumpolar Current strength variability. Nature 627, 789-796. DOI: 10.1038/s41586-024-07143-3.
5. Zheng, Y., Heng, P., Conte, M.H., Vachula, R.S., Huang, Y., 2019. Systematic chemotaxonomic profiling and novel paleotemperature indices based on alkenones and alkenoates: Potential for disentangling mixed species input. Organic Geochemistry 128, 26-41. DOI: 10.1016/j.orggeochem.2018.12.008.
6. Guitián, J., Stoll, H.M., 2021. Evolution of Sea Surface Temperature in the Southern Mid-latitudes From Late Oligocene Through Early Miocene. Paleoceanography and Paleoclimatology 36, e2020PA004199. DOI: 10.1029/2020PA004199.
